# Increased Risk of Generalized Anxiety Disorder According to Frequent Sedentary Times Based on the 16th Korea Youth Risk Behavior Web-Based Survey

**DOI:** 10.3390/children9101548

**Published:** 2022-10-12

**Authors:** Hongsun Song, Kihyuk Lee

**Affiliations:** 1 Department of Sports Science, Korea Institute of Sport Science, Seoul 01794, Korea; 2 Department of Sports Science Convergence, Dongguk University, Seoul 04620, Korea

**Keywords:** adolescents, anxiety, generalized anxiety disorder-7, sedentary behaviors, sedentary time, physical activity

## Abstract

This study aimed to identify the association between sedentary behavior and anxiety disorders in 53,510 Korean adolescents. It analyzed data from the 16th (2020) Korea Youth Risk Behavior Web-based Survey (KYRBS). The dependent variable was the generalized anxiety disorder-7 (GAD-7). The GAD-7 scores were divided into normal, mild, moderate, and severe levels. The independent variables were sedentary time for learning, other sedentary times, total sedentary time, and regular physical activity. The confounding variables were sex, grade, stress, depression, substance abuse, suicidal thoughts, violent victimization, drinking, smoking, sleep satisfaction, and smartphone addiction. A chi-squared test, one-way analysis of variance, and logistic regression analysis were used for analysis. After adjusting for all confounding variables, the risk of severe level in GAD-7 increased by 1.045 times each time the sedentary time for learning based on increased by one hour. In other sedentary time and total sedentary time, the risk of severe level in GAD-7 increased by 1.025 times and 1.045 times per hour, respectively. However, in regular physical activity, after adjusting for the confounding variables, there was no significant association with the GAD-7 levels. Therefore, to prevent generalized anxiety disorders in Korean adolescents, it is necessary to reduce the overall sedentary times including sedentary time for learning.

## 1. Introduction

Anxiety disorders refer to an abnormal condition resulting in symptoms such as tension, panic, fear, and a rapid heartbeat [1]. Continuous concern and apprehension are major symptoms of anxiety disorders that not only make it difficult to control oneself but also negatively affect normal daily life, which develops into a medical condition that requires treatment [2]. Anxiety disorders are common mental disorders that occur in adolescence and have several types. Typical anxiety disorders include generalized anxiety, panic, separation anxiety, and social anxiety disorders [3]. The prevalence of all types of anxiety disorders was surveyed in 10,123 adolescents aged 13 to 18 years across the United States, and 31.9% of them were reported to have anxiety disorders [4].

Generalized anxiety disorder is characterized by continuous and excessive anxiety and anxiety about activities or events that include daily problems and may also occur with other anxiety disorders or depression [3]. According to a survey in 2020, 11.9% of Korean adolescents had generalized anxiety disorder of moderate intensity or higher [5]. Meanwhile, previous studies have shown that anxiety disorders, including generalized anxiety disorder, are associated with all forms of mental illness, and anxiety disorders in adolescence are highly likely to occur as the first symptom of various other psychopathologies [2,6,7].

Despite the risk of anxiety disorders, the diagnosis of anxiety disorders in adolescents is insufficient or lightly dismissed [8]. It is difficult to distinguish between anxiety disorders and fear and concern in the normal developmental process [9]. Moreover, anxiety disorders progress gradually and can develop into chronic anxiety as well as worsening symptoms because of stress factors such as studies and friends [10]. Nevertheless, anxiety disorders are difficult to diagnose and treat; and according to previous studies, approximately 18% of adolescents with anxiety disorders are reported to receive treatment [11]. Moreover, Kessler et al. [7]. reported that approximately 75% of adults with chronic mental health problems develop anxiety disorders before the age of 24, which is likely to require prevention and management of early anxiety disorders.

Many previous studies have reported on the prevention and management of anxiety disorders. It is not surprising that stress, smoking, drinking, and substance use are factors that affect anxiety disorders [12,13,14,15]. A previous study has shown that stress increased the risk of severe level of GAD-7 by 26.39 times, smoking by 2.08 times, drinking by 1.91 times, and substance abuse by 10.89 times [16]. According to Härter et al. [17], various diseases such as heart disease and high blood pressure are related to anxiety disorders, including generalized anxiety disorder. Meanwhile, sedentary behavior is reported to be highly related to mental diseases [18] and chronic diseases such as obesity, high blood pressure, and hyperlipidemia.

Previous studies have reported that sedentary behavior and physical activity are related to anxiety disorders. A previous study examined the effect of sedentary time on depression, anxiety, and stress symptoms in an average 58-year-old in Australia, and overall sedentary time was significantly associated with more severe depression (coefficient [b]:0.01, 95% confidence [CI]:0.00–0.02) and anxiety (b:0.03, CI:0.02–0.04) [19]. Similarly, in adults with chronic diseases in Thailand and Vietnam, a sitting time of more than 8 h per day significantly increased anxiety disorders (odds ratio [OR]:1.42, CI:1.08–1.86) and depression (OR:1.40, CI:1.11–1.76), but high-intensity physical activity significantly decreased anxiety disorders (OR:0.58, CI:0.43–0.78) and depression (OR:0.46, CI:0.30–0.58) [20]. However, this is difficult to apply to Korean adolescents because most previous studies on the relationship between sedentary behavior and anxiety disorders have been conducted on adults.

Meanwhile, another previous study reported that anxiety disorders in Korean adolescents are related to other mental health conditions and health risk behaviors using the KYRBS [16,21]; however, no studies have been conducted on the effect of sedentary behaviors, such as sedentary time and physical activities, on anxiety disorders in Korean adolescents. Therefore, it is necessary to explain the effect of Korean adolescents’ sedentary behavior on anxiety disorders by considering various variables. Accordingly, we conducted an analysis by selecting variables affecting generalized anxiety disorder as confounding variables by referring to previous studies [12,13,14,15,16,21]. This study aimed to present valuable data to prevent anxiety disorders by highlighting the association between sedentary behaviors and anxiety disorders in Korean adolescents.

## 2. Materials and Methods

### 2.1. Participants and Procedure of Data Collection

**Figure 1:**
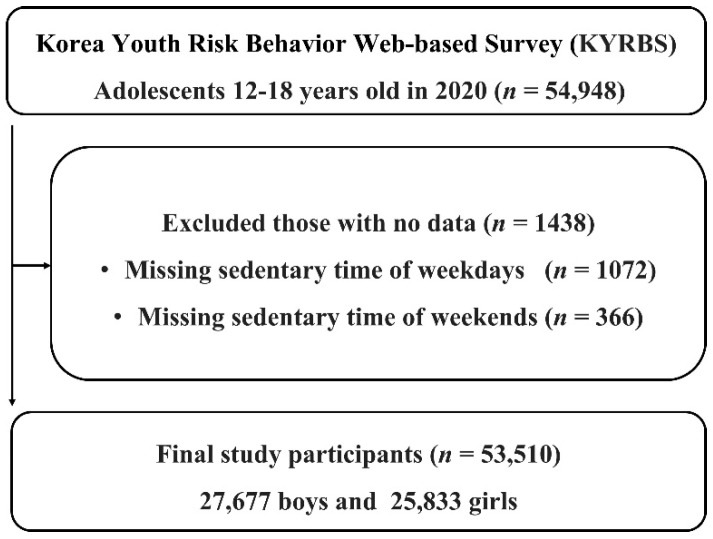
A flow diagram of the study participants.

The participants of this study were Korean adolescents. Their ages ranged from 12 to 18, and the average age of boys was 15.11 (±1.756) years, while the average age of girls was 15.07 (±1.753) years. This study used the 16th KYRBS conducted by the Korea Disease Control and Prevention Agency (KDCA). The KYRBS is an anonymous online survey conducted in 2005 to understand the health behaviors of Korean adolescents. The survey was a government-approved statistical survey (approval number 117058). The 16th KYRBS period lasted from 3 August–13 November 2020. A total of 57,925 students from 800 schools (400 middle and 400 high schools) participated. Finally, 54,948 students participated, showing a 94.9% participation rate; however, in the case of analysis related to sedentary time, 1438 individuals who did not respond were excluded. A detailed flow diagram of the study is shown in Figure 1.

### 2.2. Measurement Variables

#### 2.2.1. Independent Variable

The independent variables were sedentary time and regular physical activity. Sedentary time was defined as sedentary time for learning, other sedentary times, and total sedentary time. Sedentary time for learning included school and academic classes, watching TV or using a computer to do homework or study, and educational broadcasting. Other sedentary times included watching TV, playing games, using the Internet, chatting, and sitting on the go. The total sedentary time was the sum of the sedentary time for learning and other sedentary times. The following formula was used in this study to calculate each sedentary time for seven days: ([weekdays sedentary time × 5] + [weekends sedentary time × 2])/7. Regular physical activity was classified as “yes” and “no”, where students who completed one or more of the following three physical activities were classified as “yes”: more than five days of moderate physical activity, more than three days of vigorous physical activity, or more than three days of muscular exercise. Moderate physical activity was defined as performing physical activity with an increased heart rate than usual for more than 60 min per day, and high-intensity physical activities were defined as performing physical activities such as jogging, soccer, and hiking for more than 20 min. Muscular exercises were defined as push-ups, sit-ups, weightlifting, dumbbells, pull-ups, and parallel bars.

#### 2.2.2. Dependent Variable

The dependent variable was the general anxiety disorder-7 (GAD-7). It is a widely used evaluation tool that can effectively determine anxiety disorders in a short time and is reported to have a high correlation with anxiety symptoms evaluated by clinicians [22]. It is a self-report measurement tool that checks seven items related to anxiety with scores ranging from 0 to 3 points. In each of the seven questions, the total score of the GAD-7 was up to 21 points (0–4, normal; 5–9, mild; 10–14, moderate; and 15–21, severe anxiety disorder) [23]. A study of Finnish adolescents reported that GAD-7 reliability is 0.89 (Cronbach’s ⍺) [24].

#### 2.2.3. Confounding Variables

Potential confounding variables were selected as factors that could affect anxiety disorders. Sex, school grade, stress, depression, substance abuse, suicidal thoughts, violent victimization, drinking, smoking, sleep satisfaction, and smartphone addiction were analyzed. Sex was used without reconstruction as a confounding variable. School grades were divided into middle and high schools. The question about stress was “How much stress do you usually feel?” The five responses to stress were reconstructed as low, moderate, and high. The question that evaluated depression was “Have you ever been sad or desperate enough to stop your routine for more than two weeks in the last 12 months?” The question concerning substance abuse was “Except for therapeutic purposes, have you ever used any medications or substances ever or habitually?” The question about suicidal thoughts was “Have you seriously considered suicide in the last 12 months?” Responses to depression, substance abuse, and suicidal thoughts were “yes” or “no.” The question on violent victimization was “Have you been treated in a hospital for violence (physical assault, intimidation, or bullying) by friends, seniors, or adults in the last 12 months?” The response was re-divided into “no” for 0 and “yes” for more than 1 response. The question about drinking was “In the last 30 days, how many days did you drink more than one cup?” and the question about smoking was “In the last 30 days, how many days did you usually smoke one or more cigarettes?” Responses to these questions were reconstructed as “yes” or “no.” In the case of sleep satisfaction, the question was “Do you think the time you slept in the last seven days is enough to recover from fatigue?” The answers were reconstructed as “yes” or “no.” Smartphone addiction was evaluated on a four-point Likert scale. It consisted of 10 questions, with a total score of 40. The item scores were summed and classified into general user groups, with a total score of 22 or less. A total score of 23–30 was classified as the potential-risk user group, and a total score of 31 points or more was classified as the high-risk user group.

### 2.3. Statistical Analysis

The KYRBS was conducted with a complex sample design. The data in this study were analyzed by applying strata, clusters, weights, and finite population corrections. All data were analyzed using SPSS 25.0 (SPSS Inc., Chicago, IL, USA). The chi-square test (Rao-Scott) was used to investigate the differences in subjects’ confounding variables and physical activity according to the level of anxiety disorders. The difference between each sedentary time according to the level of anxiety disorders was analyzed using one-way ANOVA. Complex sample logistic regression analysis was conducted to analyze the association between sedentary behavior and the level of anxiety disorders. The odds ratio (OR) and 95% confidence interval (CI) for each sedentary time and regular physical activity were calculated for the level of anxiety disorders. The level of statistical significance (α) was set at 0.05.

## 3. Results

### 3.1. Characteristics of Korean Adolescents According to the Level of GAD-7

The total number of subjects in this study was 53,510, and according to the level of GAD-7, 35,795 were normal, 11,821 were mild, 4020 were moderate, and 1874 were severe. Table 1 shows the differences in the general characteristics of Korean adolescents according to their level of GAD-7. All of the general characteristics showed significant differences in the GAD-7 levels (*p* < 0.001). The chi-square test was used to determine the relationship between confounding variables and the GAD-7 levels (sex, school grade, depression, substance abuse, suicidal thoughts, violent victimization, drinking, smoking, sleep satisfaction, smartphone addiction, and regular physical activity). All confounding variables showed significant differences with the GAD-7 level (all *p* < 0.001). Table 2 shows the detailed chi-square test results between the confounding variables and the GAD-7 levels.

### 3.2. Differences in in Each Sedentary Time According to the GAD-7 Levels

Analysis of the difference between the GAD-7 levels and each sedentary time revealed a significant difference in each sedentary time according to GAD-7 levels (all *p* < 0.001). Table 3 shows the detailed differences in each sedentary time according to the GAD-7 levels.

### 3.3. Association between Sedentary Behavior and Levels of GAD-7

Table 4, Table 5, Table 6 and Table 7 show the association between sedentary behavior and GAD-7 levels using logistic regression analysis. When all confounding variables were applied (Model 3), when sedentary time increased by 1 h, the risk of mild level increased by 1.023 times (*p* < 0.001), the risk of moderate level increased by 1.032 times (*p* < 0.001), and the risk of severe level increased by 1.045 times (*p* < 0.001) in the case of sedentary time for learning. In the case of other sedentary times, the risk of mild level increased by 1.005 times (*p* = 0.303), the risk of moderate level increased by 1.008 times (*p* = 0.223), and the risk of severe level increased by 1.025 times (*p* = 0.012) in Model 3. In the case of total sedentary time, the risk of mild level increased by 1.018 times (*p* < 0.001), the risk of moderate level increased by 1.026 times (*p* < 0.001), and the risk of severe level increased by 1.045 times (*p* < 0.001) in Model 3. In the case of regular physical activity, there was no significant difference in Model 3. However, the risk of mild level increased by 1.272 times (*p* < 0.001), the risk of moderate level increased by 1.269 times (*p* < 0.001), and the risk of severe level increased by 1.486 times (*p* < 0.001), according to the absence of physical activity in Model 1, which was not adjusted for confounding variables. 

## 4. Discussion

This study aimed to determine the association between sedentary behavior and generalized anxiety disorder using the KYRBS, a national statistical survey, and to provide basic data for the development of intervention programs for the prevention and reduction in generalized anxiety disorder. The levels of generalized anxiety disorder were analyzed using the widely used GAD-7. The proportion of patients with generalized anxiety disorder was 3.5%, 7.51%, 22.09%, and 66.89% in the severe, moderate, mild, and normal groups, respectively. In a previous study of Saudis over 18 years old, the distribution of levels of GAD-7 was found to be normal 37.9%, mild 33.10%, severe 15.7%, and severe 13.3%, respectively [25]. A previous study divided the level of GAD-7 in the same way as our study, and the proportion of severe was higher than that of our study. Therefore, the level of GAD-7 may vary by country and race, and the results need to be analyzed in consideration of this. The GAD-7 levels showed significant differences according to sex, school grade, stress, depression, substance abuse, suicidal thoughts, violent victimization, drinking, smoking, sleep satisfaction, smartphone addiction, and total sedentary time. There was a significant difference in the GAD-7 levels according to sedentary behavior.

As a result of cross-analysis by selecting confounding variables that were judged to affect the GAD-7, all confounding variables showed significant differences from the GAD-7. By sex, female adolescents had a higher percentage of severe symptoms at the GAD-7 level than male adolescents (4.9% vs. 2.2%). This is similar to the results of previous studies that compared differences in anxiety between men and women [26]. In addition, all other confounding variables were found to be related to the GAD-7 in previous studies [12,13,14,15,16,21].

Accordingly, we conducted a logistic regression analysis by applying all of the selected confounding variables and found a close association between sedentary behavior and the GAD-7 levels. Each sedentary time showed a significant difference according to the GAD-7 levels. In addition, in the case of logistic regression analysis in Model 3, considering all confounding variables, the probability of being included in the severity level of the GAD-7 increased by 1.045 times as the learning time increased by 1 h, and the other sedentary times increased by 1.058 times. In addition, as the total sedentary time increased by 1 h, the probability of inclusion in the severity level of the GAD-7 increased by 1.045 times. Previous studies have reported a close association between sedentary behavior and anxiety in adults; in particular, sedentary time over 8 h has significantly increased anxiety and sedentary time [20]. Previous studies have verified the relationship between high sedentary behavior and anxiety in adults [19,20,27]; however, our study is meaningful in that sedentary behavior increases the risk of anxiety disorders in adolescents. Moreover, the results of this study support the previous study that the presence or absence of sedentary behavior increases the probability of serious symptoms of depression and anxiety by 1.31 times for adolescents aged 14 to 15 years in Canada [28]. However, Bélair et al. (2018) did not separately classify anxiety and depression. They identified an association between sedentary behavior and symptoms of depression and anxiety in the same category. In addition, they categorized sedentary behavior and analyzed the behavior of watching TV or DVD or playing games for more than 2 h per day by classifying it into sedentary behavior. However, we obtained similar results to those of a previous study by dividing sedentary behaviors into sedentary time for learning, other sedentary times, and total sedentary time on an hourly basis. As adolescents are more exposed to the risk of anxiety disorders than adults, and anxiety disorders in adolescence are likely to lead to adulthood [7], a reduction in sedentary time can help prevent anxiety disorders.

Regular physical activity or moderate exercise is also known to help alleviate anxiety disorders [29,30]; however, differing from sedentary hours in this study, regular physical activity was not significantly related to the GAD-7 levels in Korean adolescents. In Model 1, which does not consider confounding variables, the absence of regular physical activity increased the possibility of conversion to the severity the GAD-7 level by 1.439 times, but there was no significant relationship in Model 2, which considers confounding variables. However, considering previous studies that showed that the higher the intensity of physical activity, the lower the probability of developing anxiety disorders [20,31], it is necessary to check the association between physical activity and anxiety disorders through various methods such as intensity and type of physical activity.

This study is meaningful in that it uses large-scale data and analyzes the association between generalized anxiety disorder and sedentary behavior among Korean adolescents in consideration of various confounding variables that can affect anxiety disorders. Although previous studies have generally reported that stress negatively affects generalized anxiety disorder [32,33,34], no studies have reported that sedentary time for learning directly affects generalized anxiety disorder. We assumed that learning-induced stress could be one of the causes of generalized anxiety disorders, whereas other sedentary time could be different. In addition, as shown in our study results, sedentary time for learning had a higher odds ratio for the risk of anxiety disorders than other sedentary time (1.045 times vs. 1.025 times). This suggests a new approach that sedentary time for learning among various sedentary behaviors can increase the risk of generalized anxiety disorder. Moreover, we used the GAD-7 to evaluate generalized anxiety disorder, but it has also been reported that it can be utilized for the evaluation of panic, social anxiety, and post-traumatic stress disorders [35]. Therefore, the GAD-7 can be used to evaluate overall anxiety disorders; again, the association between sedentary time and overall anxiety disorders has been firmly demonstrated in adolescents.

The limitations of this study are as follows. First, the KYRBS was conducted online as a self-report survey written by adolescents. It is an anonymous survey; however, respondents are likely to have reported reduced health-related behaviors (sexual behavior, smoking, drinking, violence, or suicide) out of fear of revealing their behavioral characteristics. Second, it is possible that the results of confounding variables did not accurately reflect the status of adolescents as a subjective response. Finally, as a cross-sectional study, this study could not clearly explain the association between generalized anxiety disorder and sedentary behavior. Therefore, as a follow-up study, it is necessary to examine the effect of intervention programs, such as exercise, on generalized anxiety disorder.

## 5. Conclusions

As sedentary behavior increased, the level of generalized anxiety disorder also significantly increased. In the case of sedentary time for learning and total sedentary time, the level of generalized anxiety disorder increased, even when confounding variables such as sex, school grade, stress, depression, substance abuse, suicidal thoughts, violent victimization, drinking, smoking, sleep satisfaction, smartphone addiction, and regular physical activity were considered. Reducing total sedentary time, including sedentary time for learning, can help manage generalized anxiety disorder in Korean adolescents.

## Figures and Tables

**Table 1:** General characteristics of the Korean adolescents according to level of GAD-7.

Variables	Normal (*n* = 35,795)	Mild (*n* = 11,821)	Moderate (*n* = 4020)	Severe (*n* = 1874)	Total (*n* = 53,510)	x^2^ (*p*)
Sex						
Male	20,394 (73.7)	5156 (18.7)	1515 (5.4)	612 (2.2)	27,677 (100.0)	420.779
Female	15,401 (59.5)	6665 (26.0)	2505 (9.7)	1262 (4.9)	25,833 (100.0)	<0.001
School grade						
Middle school	19,576 (69.3)	5767 (20.6)	1974 (7.0)	869 (3.1)	28,186 (100.0)	57.467
High school	16,219 (63.6)	6054 (24.3)	2046 (8.1)	1005 (4.0)	25,324 (100.0)	<0.001
Economic status						
High	4399 (75.5)	944 (16.1)	317 (5.2)	189 (3.3)	5849 (100.0)	148.801
Middle	27,573 (67.5)	8988 (22.4)	2883 (7.1)	1221 (3.0)	40,665 (100.0)	<0.001
Low	3823 (53.9)	1889 (27.3)	820 (12.1)	464 (6.8)	6996 (100.0)	
Health status						
High	27,637 (73.7)	7059 (19.1)	2010 (5.3)	738 (2.0)	37,444 (100.0)	687.100
Middle	6660 (54.9)	3476 (28.9)	1316 (11.2)	584 (5.0)	12,036 (100.0)	<0.001
Low	1498 (36.3)	1286 (33.1)	694 (16.9)	552 (13.6)	4030 (100.0)	

Values are *n* (%). Percentage is weighted.

**Table 2:** Differences in health risk behaviors according to level of GAD-7.

Variables	Normal (*n* = 35,795)	Mild (*n* = 11,821)	Moderate (*n* = 4020)	Severe (*n* = 1874)	Total (*n* = 53,510)	x^2^ (*p*)
Stress						
Low	10,896 (93.8)	607 (5.1)	101 (0.8)	31 (0.3)	11,635 (100.0)	2214.462
Moderate	17,961 (75.3)	4812 (20.5)	804 (3.4)	202 (0.9)	23,779 (100.0)	<0.001
High	6938 (37.9)	6402 (35.8)	3115 (17.2)	1641 (9.1)	18,096 (100.0)	
Depression						
No	31,075 (77.1)	7018 (17.8)	1548 (3.9)	453 (1.1)	40,094 (100.0)	3206.397
Yes	4720 (35.1)	4803 (35.9)	2472 (18.3)	1421 (10,7)	13,416 (100.0)	<0.001
Suicidal thoughts						
No	34,334 (71.7)	9838 (20.9)	2670 (5.6)	861 (1.8)	47,703 (100.0)	2784.583
Yes	1461 (25.0)	1983 (34.0)	1350 (23.6)	1013 (17.4)	5807 (100.0)	<0.001
Violent victimization						
No	35,482 (66.9)	11,655 (22.3)	3923 (7.4)	1765 (3.4)	52,825 (100.0)	117.952
Yes	313 (45.6)	166 (25.2)	97 (13.8)	109 (15.4)	685 (100.0)	<0.001
Drinking						
No	32,610 (67.9)	10,272 (21.6)	3405 (7.3)	1531 (3.2)	47,818 (100.0)	117.181
Yes	3185 (56.1)	1549 (27.1)	615 (10.8)	343 (5.9)	5692 (100.0)	<0.001
Smoking						
No	32,618 (67.6)	10,448 (22.0)	3461 (7.2)	1552 (3.2)	48,079 (100.0)	45.026
Yes	3177 (57.8)	1373 (25.5)	559 (10.5)	322 (6.2)	5431 (100.0)	<0.001
Substance abuse						
No	35,655 (66.9)	11,711 (22.3)	3963 (7.4)	1798 (3.4)	53,127 (100.0)	114.178
Yes	140 (38.2)	110 (27.1)	57 (15.6)	76 (19.1)	383 (100.0)	<0.001
Sleep satisfaction						
No	22,552 (60.4)	9451 (25.9)	3444 (9.3)	1646 (4.4)	37,093 (100.0)	668.289
Yes	13,243 (80.7)	2370 (14.3)	576 (3.5)	228 (1.5)	16,417 (100.0)	<0.001
Smartphone addiction						
General	29,405 (73.2)	7531 (18.9)	2247 (5.6)	944 (2.3)	40,127 (100.0)	649.253
Potential-risk	5884 (49.2)	3836 (32.7)	1453 (12.3)	653 (5.7)	11,826 (100.0)	<0.001
High-risk	506 (31.8)	454 (30.3)	320 (20.5)	277 (17.4)	1557 (100.0)	
Regular physical activity						
No	20,702 (64.4)	7547 (23.6)	2558 (8.1)	1258 (3.9)	32,065 (100.0)	57.107
Yes	15,093 (70.1)	4274 (20.1)	1462 (6.9)	616 (2.9)	21,445 (100.0)	<0.001

**Table 3:** Difference in each sedentary time according to level of GAD-7.

	Normal	Mild	Moderate	Severe	*p*
Sedentary time for learning (hours/day)	5.669 ± 0.024	6.135 ± 0.038	6.289 ± 0.058	6.329 ± 0.089 ^a,b,c^	<0.001
Other sedentary time (hours/day)	4.212 ± 0.016	4.367 ± 0.027	4.496 ± 0.045	4.674 ± 0.078 ^d,e,f^	<0.001
Total sedentary time (hours/day)	9.882 ± 0.025	10.502 ± 0.042	10.785 ± 0.065	11.003 ± 0.096 ^g,h,i^	<0.001

^a^: *p* < 0.001 (vs. normal); ^b^: *p* = 0.035 (vs. mild); ^c^: *p* = 0.700 (vs. moderate); ^d^: *p* < 0.001 (vs. normal); ^e^: *p* < 0.001 (vs. mild); ^f^: *p* = 0.045 (vs. moderate); ^g^: *p* < 0.001 (vs. normal); ^h^: *p* < 0.001 (vs. mild); ^i^: *p* = 0.059 (vs. moderate).

**Table 4:** Logistic regression analysis to level of GAD-7 based on sedentary time for learning.

		Model 1		Model 2		Model 3	
Sedentary time for learning		OR (95% CI)	*p*	OR (95% CI)	*p*	OR (95% CI)	*p*
Level of GAD-7	Normal	Reference	-	Reference	-	Reference	-
	Mild	1.041 (1.035–1.048)	<0.001	1.029 (1.023–1.035)	<0.001	1.023 (1.016–1.030)	<0.001
	Moderate	1.055 (1.045–1.065)	<0.001	1.036 (1.025–1.047)	<0.001	1.032 (1.021–1.042)	<0.001
	Severe	1.059 (1.043–1.074)	<0.001	1.041 (1.025–1.058)	<0.001	1.045 (1.029–1.061)	<0.001

OR: odds ratio; Model 1 was not adjusted; Model 2 was adjusted for gender, school grade, and regular physical activity; Model 3 was adjusted for gender, school grade, stress, depression, substance abuse, suicidal thoughts, violent victimization, drinking, smoking, sleep satisfaction, smartphone addiction, and regular physical activity.

**Table 5:** Logistic regression analysis to level of GAD-7 based on other sedentary time.

		Model 1		Model 2		Model 3	
Other sedentary time		OR (95% CI)	*p*	OR (95% CI)	*p*	OR (95% CI)	*p*
Level of GAD-7	Normal	Reference	-	Reference	-	Reference	-
	Mild	1.020 (1.013–1.028)	<0.001	1.020 (1.012–1.028)	<0.001	1.005 (0.996–1.013)	0.303
	Moderate	1.036 (1.025–1.047)	<0.001	1.035 (1.023–1.046)	<0.001	1.008 (0.995–1.021)	0.223
	Severe	1.056 (1.039–1.074)	<0.001	1.063 (1.044–1.081)	<0.001	1.025 (1.006–1.045)	0.012

OR: odds ratio; Model 1 was not adjusted; Model 2 was adjusted for gender, school grade, and regular physical activity; Model 3 was adjusted for gender, school grade, stress, depression, substance abuse, suicidal thoughts, violent victimization, drinking, smoking, sleep satisfaction, smartphone addiction, and regular physical activity.

**Table 6:** Logistic regression analysis to level of GAD-7 disorder based on total sedentary time.

		Model 1		Model 2		Model 3	
Total sedentary time		OR (95% CI)	*p*	OR (95% CI)	*p*	OR (95% CI)	*p*
Level of GAD-7	Normal	Reference	-	Reference	-	Reference	-
	Mild	1.038 (1.033–1.044)	<0.001	1.030 (1.024–1.035)	<0.001	1.018 (1.012–1.024)	<0.001
	Moderate	1.056 (1.048–1.064)	<0.001	1.042 (1.033–1.051)	<0.001	1.026 (1.017–1.035)	<0.001
	Severe	1.069 (1.057–1.082)	<0.001	1.062 (1.047–1.076)	<0.001	1.045 (1.031–1.059)	<0.001

OR: odds ratio; Model 1 was not adjusted; Model 2 was adjusted for gender, school grade, and regular physical activity; Model 3 was adjusted for gender, school grade, stress, depression, substance abuse, suicidal thoughts, violent victimization, drinking, smoking, sleep satisfaction, smartphone addiction, and regular physical activity.

**Table 7:** Logistic regression analysis to level of GAD-7 based on regular physical activity.

		Model 1		Model 2		Model 3	
Regular physical activity		OR (95% CI)	*p*	OR (95% CI)	*p*	OR (95% CI)	*p*
Level of GAD-7	Normal	Reference	-	Reference	-	Reference	-
	Mild	1.272 (1.213–1.333)	<0.001	1.046 (0.998–1.097)	0.063	1.026 (0.974–1.080)	0.335
	Moderate	1.269 (1.182–1.364)	<0.001	0.949 (0.884–1.018)	0.145	0.945 (0.871–1.026)	0.177
	Severe	1.486 (1.338–1.649)	<0.001	1.033 (0.919–1.162)	0.588	1.058 (0.932–1.202)	0.382

OR: odds ratio; Model 1 was not adjusted; Model 2 was adjusted for gender, school grade, and total sedentary time; Model 3 was adjusted for gender, school grade, stress, depression, substance abuse, suicidal thoughts, violent victimization, drinking, smoking, sleep satisfaction, smartphone addiction, and total sedentary time.

## Data Availability

Releasing of the data by the researcher is not legally permitted. All data are available from the database of the KDCA.

## References

[1] Kieling C., Baker-Henningham H., Belfer M., Conti G., Ertem I., Omigbodun O., Rohde L.A., Srinath S., Ulkuer N., Rahman A. (2011). Child and adolescent mental health worldwide: Evidence for action. Lancet.

[2] Shear M.K., Bjelland I., Beesdo K., Gloster A.T., Wittchen H.U. (2007). Supplementary dimensional assessment in anxiety disorders. Int. J. Methods Psychiatr. Res..

[3] American Psychiatric Association (2013). Diagnostic and Statistical Manual of Mental Disorders: DSM-5.

[4] Merikangas K.R., He J.P., Burstein M., Swanson S.A., Avenevoli S., Cui L., Benjet C., Georgiades K., Swendsen J. (2010). Lifetime prevalence of mental disorders in U.S. adolescents: Results from the National Comorbidity Survey Replication-Adolescent Supplement (NCS-A). J. Am. Acad. Child. Adolesc. Psychiatry.

[5] Korea Disease Control and Prevention Agency 2020 Reports on the Korea Youth Risk Behavior Web-Based Survey. https://www.kdca.go.kr/yhs/.

[6] Wittchen H.U., Lieb R., Pfister H., Schuster P. (2000). The waxing and waning of mental disorders: Evaluating the stability of syndromes of mental disorders in the population. Compr. Psychiatry.

[7] Kessler R.C., Berglund P., Demler O., Jin R., Merikangas K.R., Walters E.E. (2005). Lifetime prevalence and age-of-onset distributions of DSM-IV disorders in the National Comorbidity Survey Replication. Arch. Gen. Psychiatry.

[8] Achenbach T.M. (1993). Implications of multiaxial empirically based assessment for behavior therapy with children. Behav. Ther..

[9] Ramsawh H.J., Chavira D.A. (2016). Association of childhood anxiety disorders and quality of life in a primary care sample. J. Dev. Behav. Pediatr..

[10] Woodward L.J., Fergusson D.M. (2001). Life course outcomes of young people with anxiety disorders in adolescence. J. Am. Acad. Child. Adolesc. Psychiatry.

[11] Merikangas K.R., He J.P., Burstein M., Swendsen J., Avenevoli S., Case B., Georgiades K., Heaton L., Swanson S., Olfson M. (2011). Service utilization for lifetime mental disorders in U.S. adolescents: Results of the National Comorbidity Survey-Adolescent Supplement (NCS-A). J. Am. Acad. Child. Adolesc. Psychiatry.

[12] Smith J.P., Randall C.L. (2012). Anxiety and alcohol use disorders: Comorbidity and treatment considerations. Alcohol. Res..

[13] Smith J.P., Book S.W. (2008). Anxiety and substance use disorders: A review. Psychiatr. Times.

[14] Shin H.K., Lee M.S., Kim H.G. (2011). An Empirical Study on Mobile Usage Behavior–Focusing on Smartphone Usage Addiction. Informatiz. Policy.

[15] Mohamad N.E., Sidik S.M., Akhtari-Zavare M., Gani N.A. (2021). The prevalence risk of anxiety and its associated factors among university students in Malaysia: A national cross-sectional study. BMC Public Health.

[16] Woo K.S., Ji Y., Lee H.J., Choi T.Y. (2021). The Association of Anxiety Severity with Health Risk Behaviors in a Large Representative Sample of Korean Adolescents. Soa Chongsonyon Chongsin Uihak.

[17] Härter M.C., Conway K.P., Merikangas K.R. (2003). Associations between anxiety disorders and physical illness. Euro Arch. Psychiatry Clin. Neurosci..

[18] Teychenne M., Costigan S.A., Parker K. (2015). The association between sedentary behaviour and risk of anxiety: A systematic review. BMC Public Health.

[19] Rebar A.L., Vandelanotte C., Van Uffelen J., Short C., Duncan M.J. (2014). Associations of overall sitting time and sitting time in different contexts with depression, anxiety, and stress symptoms. Ment. Health Phys. Act..

[20] Pengpid S., Peltzer K. (2019). High sedentary behaviour and low physical activity are associated with anxiety and depression in Myanmar and Vietnam. Int. J. Env. Res. Public Health.

[21] Lim S.J. (2021). The Associated Factors with Generalized Anxiety Disorder in Korean Adolescents. Korean Public Health Res..

[22] Titov N., Andrews G., Robinson E., Schwencke G., Johnston L., Solley K., Choi I. (2009). Clinician-assisted Internet-based treatment is effective for generalized anxiety disorder: Randomized controlled trial. Aust. N. Z. J. Psychiatry.

[23] Spitzer R.L., Kroenke K., Williams J.B., Löwe B. (2006). A brief measure for assessing generalized anxiety disorder: The GAD-7. Arch. Intern. Med..

[24] Tiirikainen K., Haravuori H., Ranta K., Kaltiala-Heino R., Marttunen M. (2019). Psychometric properties of the 7-item Generalized Anxiety Disorder Scale (GAD-7) in a large representative sample of Finnish adolescents. Psychiatry Res..

[25] Aljurbua F.I., Selaihem A., Alomari N.A., Alrashoud A.M. (2021). A cross-sectional study on generalized anxiety disorder and its socio-demographic correlates among the general population in Saudi Arabia. J. Fam. Med. Prim. Care.

[26] Wittchen H.U., Nelson C.B., Lachner G. (1998). Prevalence of mental disorders and psychosocial impairments in adolescents and young adults. Psychol. Med..

[27] Allen M.S., Walter E.E., Swann C. (2019). Sedentary behavior and risk of anxiety: A systematic review and meta-analysis. J. Affect. Disord..

[28] Bélair M.A., Kohen D.E., Kingsbury M., Colman I. (2018). Relationship between leisure time physical activity, sedentary behaviour and symptoms of depression and anxiety: Evidence from a population-based sample of Canadian adolescents. BMJ Open.

[29] Stubbs B., Vancampfort D., Rosenbaum S., Firth J., Cosco T., Veronese N., Salum G.A., Schuch F.B. (2017). An examination of the anxiolytic effects of exercise for people with anxiety and stress-related disorders: A meta-analysis. Psychiatry Res..

[30] Larun L., Nordheim L.V., Ekeland E., Hagen K.B., Heian F. (2006). Exercise in prevention and treatment of anxiety and depression among children and young people. Cochrane Database Syst. Rev..

[31] Gordon B.R., McDowell C.P., Lyons M., Herring M.P. (2020). Resistance exercise training for anxiety and worry symptoms among young adults: A randomized controlled trial. Sci. Rep..

[32] White J., Keenan M., Brooks N. (1992). Stress control: A controlled comparative investigation of large group therapy for generalized anxiety disorder. Behav. Cogn. Psychother..

[33] Hoge E.A., Bui E., Marques L., Metcalf C.A., Morris L.K., Robinaugh D.J., Worthington J.J., Pollack M.H., Simon N.M. (2013). Randomized controlled trial of mindfulness meditation for generalized anxiety disorder: Effects on anxiety and stress reactivity. J. Clin. Psychiatry.

[34] Hoge E.A., Bui E., Goetter E., Robinaugh D.J., Ojserkis R.A., Fresco D.M., Simon N.M. (2015). Change in decentering mediates improvement in anxiety in mindfulness-based stress reduction for generalized anxiety disorder. Cognit. Ther. Res..

[35] Kroenke K., Spitzer R.L., Williams J.B., Monahan P.O., Löwe B. (2007). Anxiety disorders in primary care: Prevalence, impairment, comorbidity, and detection. Ann. Intern. Med..

